# Supplementary material for: Photoresponsive supramolecular coordination polyelectrolyte as smart anticounterfeiting inks
Source: Nat Commun. 2021 Mar 1;12:1363. doi: 10.1038/s41467-021-21677-4 (PMC7921134; doi:10.1038/s41467-021-21677-4)
Supplement: Supplementary file 1 — Supplementary Information [file 41467_2021_21677_MOESM1_ESM.pdf]

Supplementary Information

Photoresponsive Supramolecular Coordination Polyelectrolyte as Smart Anticounterfeiting Inks

Li et al.

Supplementary Methods

All chemicals were commercially available unless mentioned otherwise. Diethyl 4-hydroxyisopyridine (**6**) was prepared according to the reported procedure as follow.[1] Chelidamic acid hydrate (2.3 g, 12.56 mmol) was dispersed in ethanol (60 mL). Then, concentrated sulfuric acid (5 mL) was added drop by drop to the above solution. The mixture was refluxed at 90 °C for 4 h. After cooling down to room temperature, the reaction mixture was adjusted to pH 7 with saturated sodium bicarbonate aqueous solution. Ethanol was removed under vacuum. The residual solution was extracted with CH_2Cl_2 (3 × 50 mL). Then, the organic phase was concentrated and dried under vacuum. The crude product was recrystallized (1:9 ethanol/n-hexane), and compound **6** was obtained as white powder in 40% yield. 3,3'-(3,3,4,4,5,5-Hexafluoro-1-cyclopentene-1,2-diyl)bis[5-bromo-2-methylthiophene] (**3**) was purchased from Chemhui chemical company. While bis-ligand **L** was once reported,[2] the synthetic procedure we report here is different from the reported method. Thus, detailed synthetic procedure was described.

All NMR spectra were recorded on a Bruker 400 instrument at 25 °C. Elemental analysis was carried out on a Thermo Flash EA 1112 analyzer. FTIR spectra were measured by a Bruker Vector 22 spectrometer in the range of 400-4000 cm^{-1} at a resolution of 4 cm^{-1} (16 scans were collected). The Zeta potential was obtained on a Nano-ZS90 nanoparticle size and potential analyzer. The steady-state luminescence spectra were measured on an Edinburgh Instruments FS920P near-infrared spectrometer, with a 450 W xenon lamp as the steady-state excitation source, a double excitation monochromator (1800 lines·mm^{-1}), an emission monochromator (600 lines·mm^{-1}), and a semiconductor cooled Hamamatsu RMP928 photomultiplier tube. The UV-vis spectrum was recorded on an Agilent Carry 100 UV spectrometer, measured from 200 to 800 nm. Dynamic light scattering (DLS) measurements were conducted on a laser light scattering spectrometer (BI-200SM) equipped with a digital correlator at 532 nm at a scattering angle of 90° under 25 °C. The 300 nm UV light irradiation experiment was carried out under a 500W Hg lamp with 300 nm optical filter, and the visible light irradiation experiment was performed using a CEL HXF300 xenon lamp (300W) with >450nm cutoff

filter. The photographs were taken under a ZF-7A UV lamp (8 W).

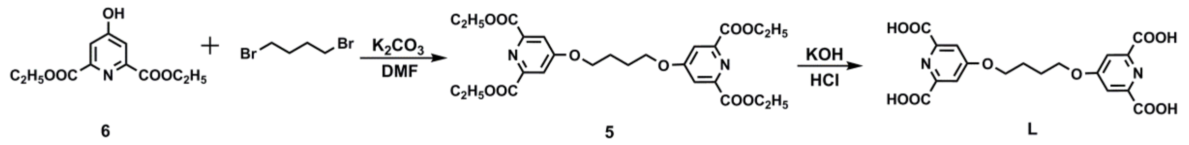

Supplementary Fig. 1 Synthetic route of compound **L**.

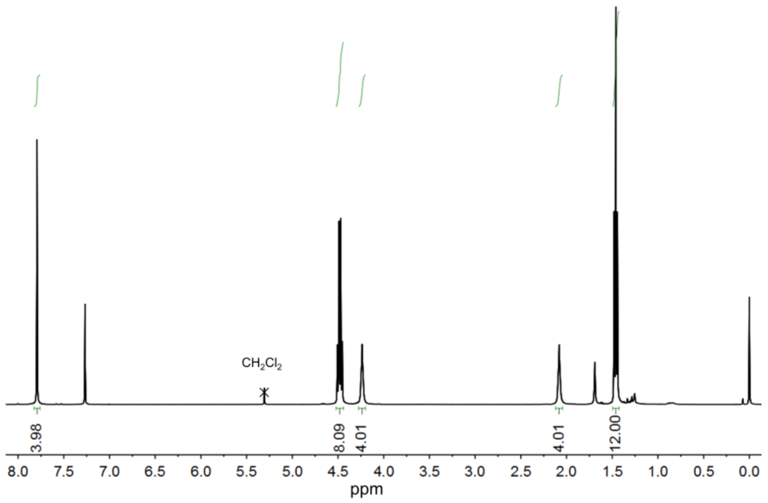

Supplementary Fig. 2 ^{1}H NMR spectrum of compound **5** ($CDCl_3$, 400 MHz, 25 °C).

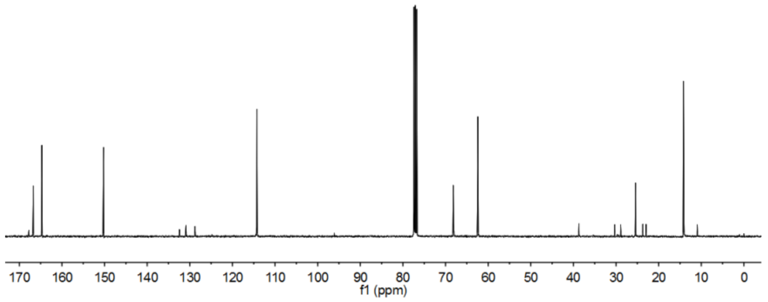

Supplementary Fig. 3 ^{13}C NMR spectrum of compound **5** ($CDCl_3$, 100 MHz, 25 °C).

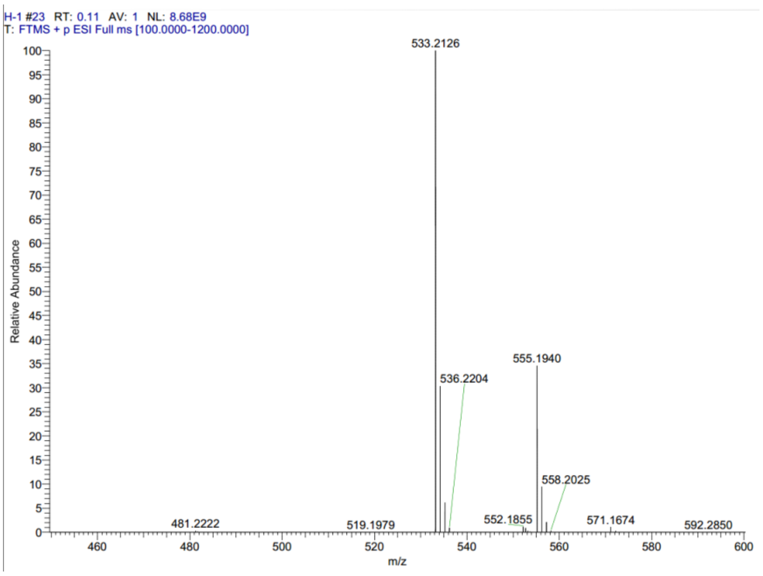

Supplementary Fig. 4 HRMS spectrum of compound **5.**

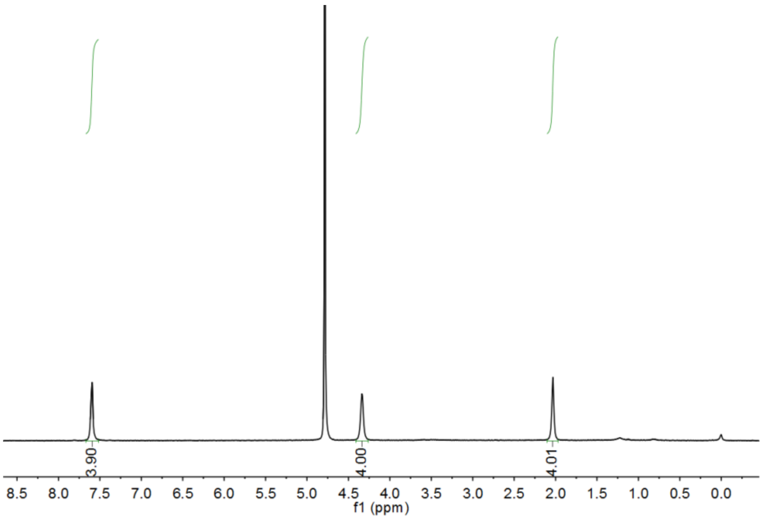

Supplementary Fig. 5 ^{1}H NMR spectrum of compound **L** (D_2O, 400 MHz, 25 °C).

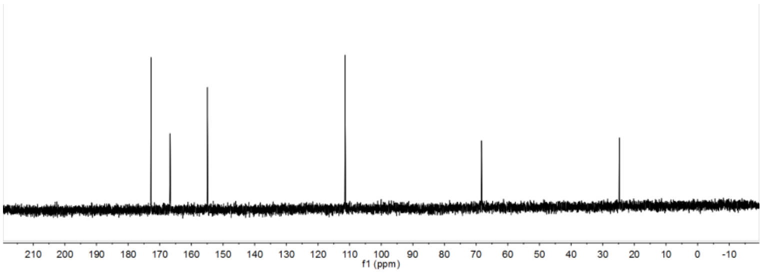

Supplementary Fig. 6 ^{13}C NMR spectrum of compound **L** (D_2O, 100 MHz, 25 °C).

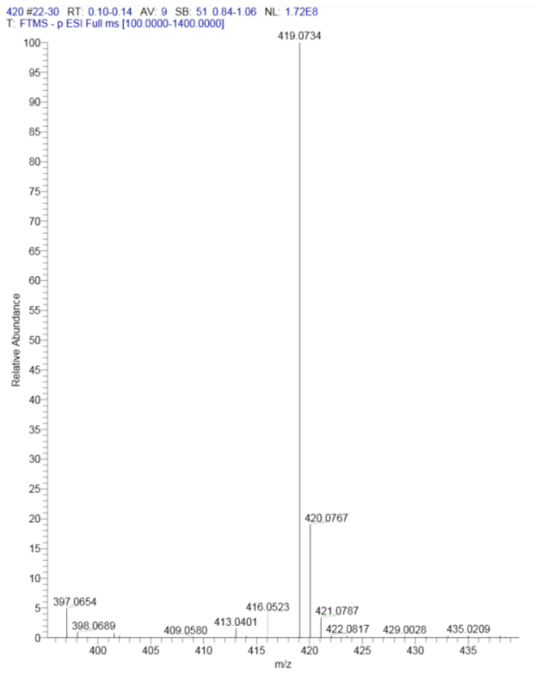

Supplementary Fig. 7 HRMS spectrum of compound **L**.

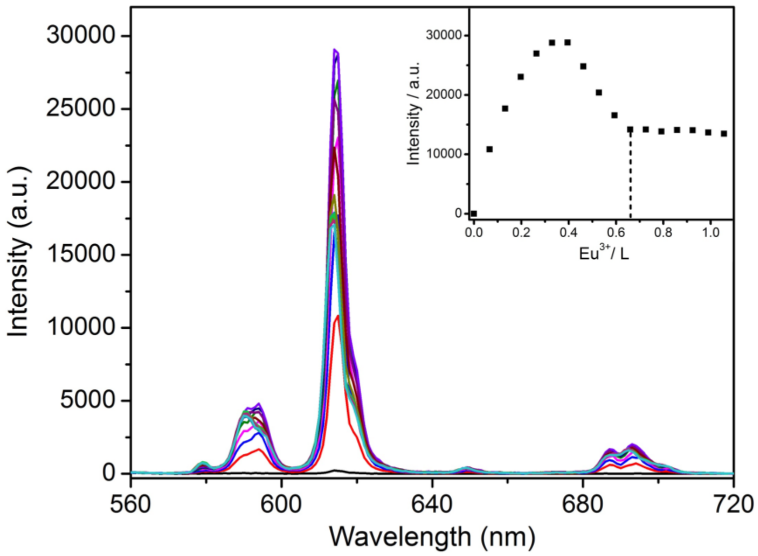

Supplementary Fig. 8 Emission changes of compound **L** upon the addition of 0-1.065 equiv. of $EuCl_3$ in H_2O at 25 °C. Inset shows corresponding emission intensity changes at 615 nm (λ_{ex} = 280 nm).

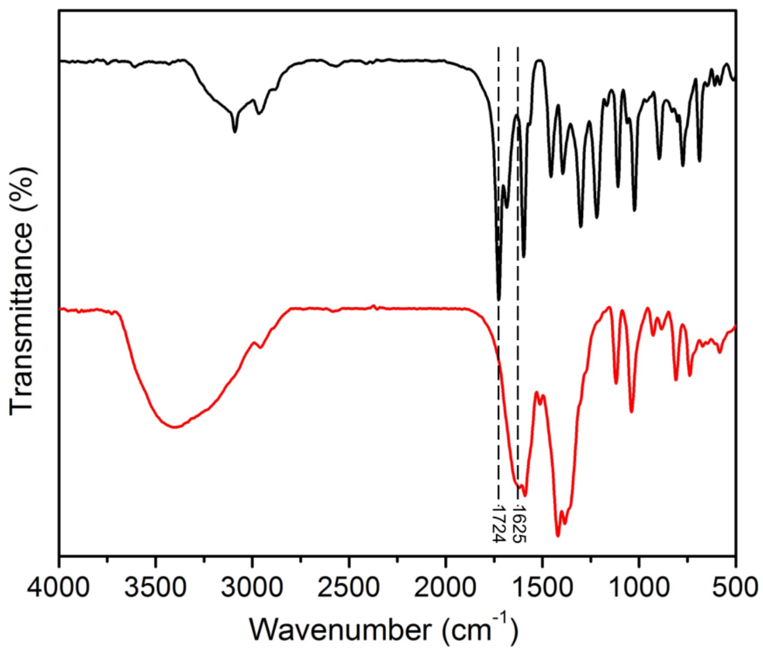

Supplementary Fig. 9 FTIR spectra of compound **L** (black curve) and Eu^{3+}-**L** (red curve).

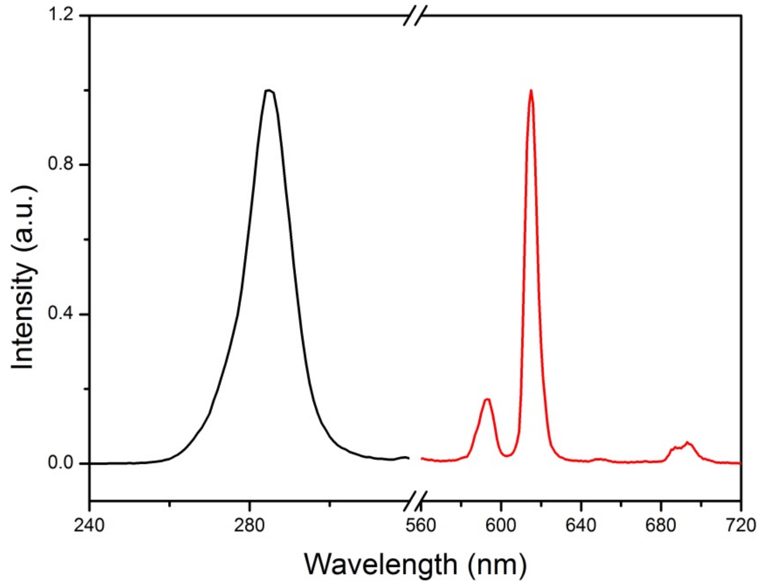

Supplementary Fig. 10 Normalized excitation (black, $\lambda_{em} = 615$ nm) and emission (red, $\lambda_{ex} = 300$ nm) spectra of Eu^{3+}-**L**.

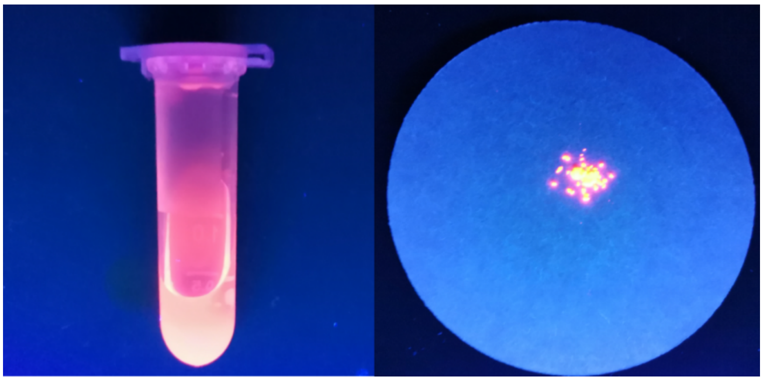

Supplementary Fig. 11 Photos of Eu^{3+}-**L** aqueous solution and powder form.

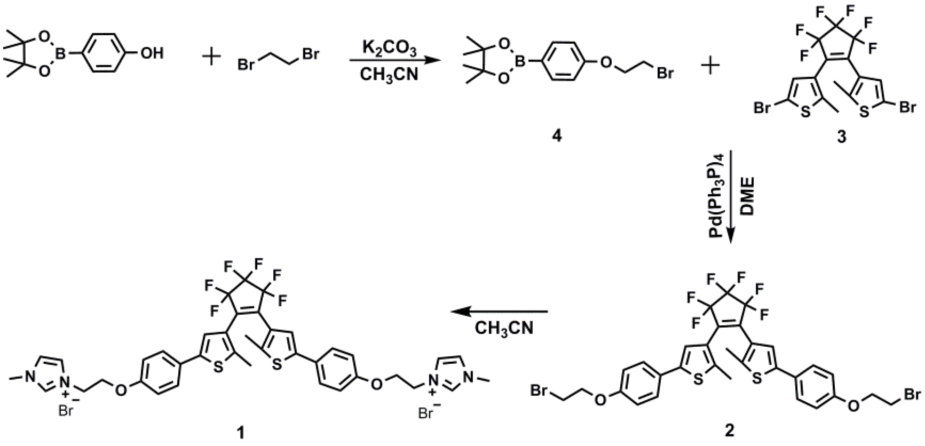

Supplementary Fig. 12 Synthetic route of compound OF-**1**.

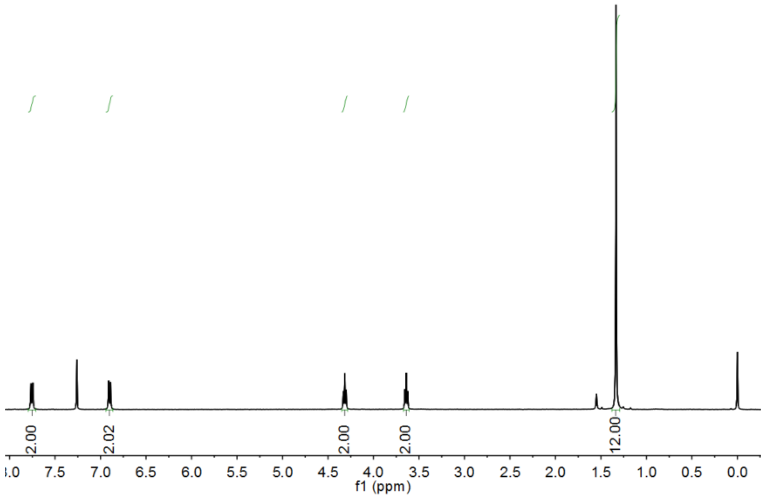

Supplementary Fig. 13 ^1H NMR spectrum of compound **4** ($CDCl_3$, 400 MHz, 25 °C).

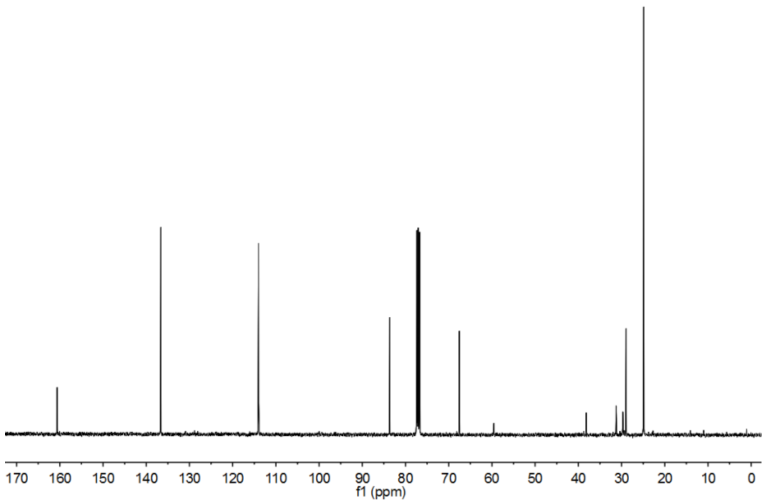

Supplementary Fig. 14 ^{13}C NMR spectrum of compound **4** ($CDCl_3$, 100 MHz, 25 °C).

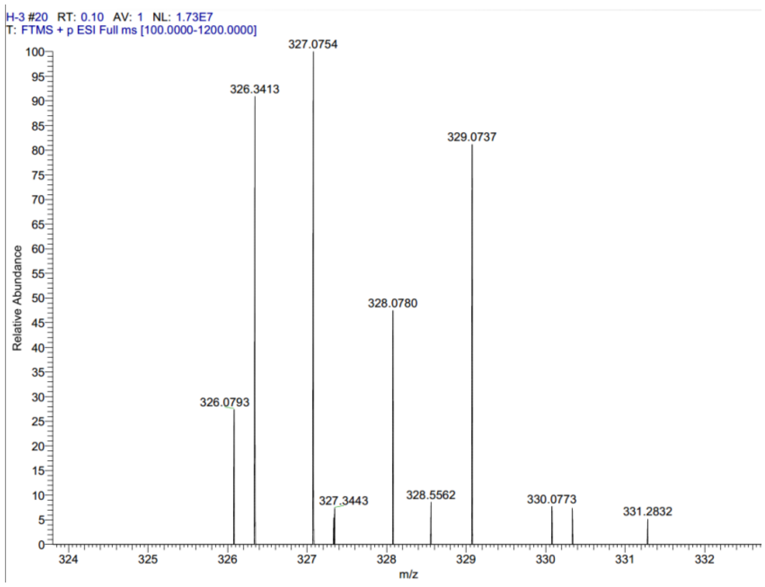

Supplementary Fig. 15 HRMS spectrum of compound **4**.

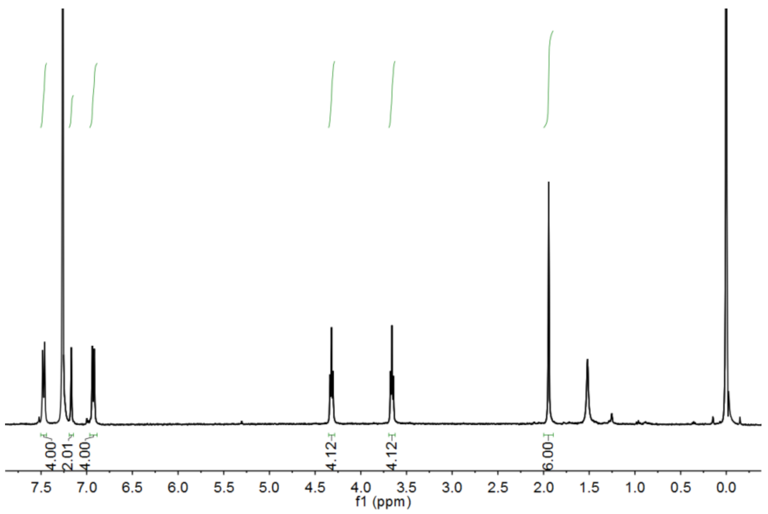

Supplementary Fig. 16 ^{1}H NMR spectrum of compound **2** ($CDCl_3$, 400 MHz, 25 °C).

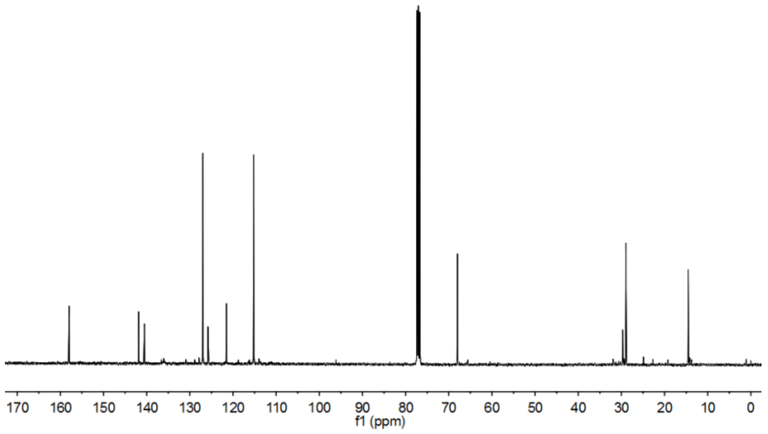

Supplementary Fig. 17 ^{13}C NMR spectrum of compound **2** ($CDCl_3$, 100 MHz, 25 °C).

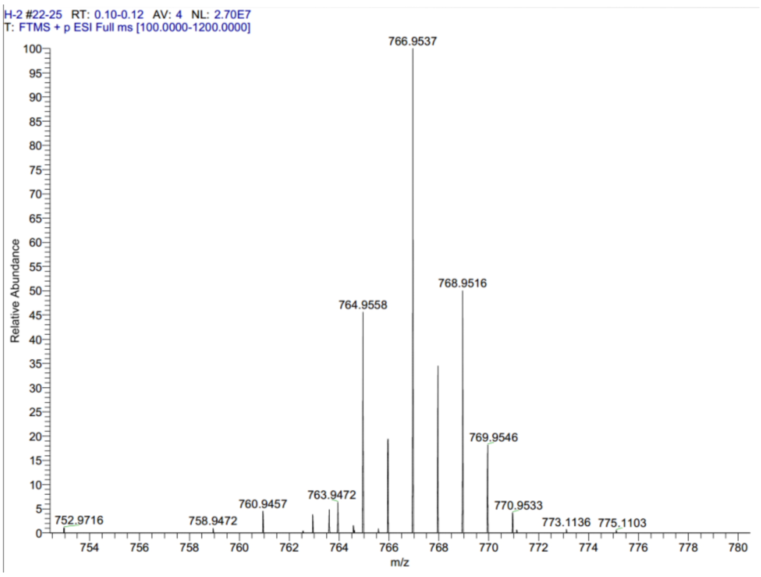

Supplementary Fig. 18 HRMS spectrum of compound **2**.

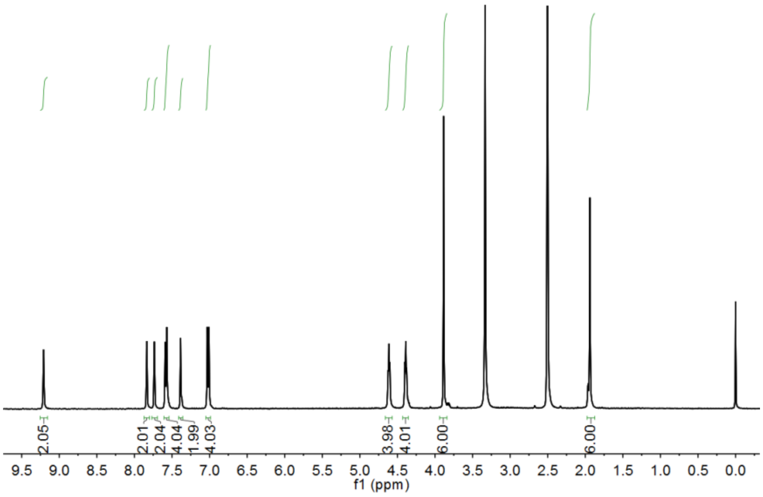

Supplementary Fig. 19 ^{1}H NMR spectrum of compound OF-**1** (DMSO-d_6, 400 MHz, 25 °C).

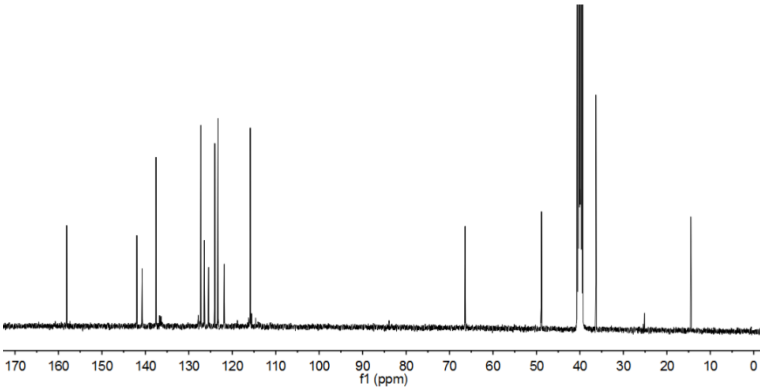

Supplementary Fig. 20 ^{13}C NMR spectrum of compound OF-**1** (DMSO-d_6, 100 MHz,

25 °C).

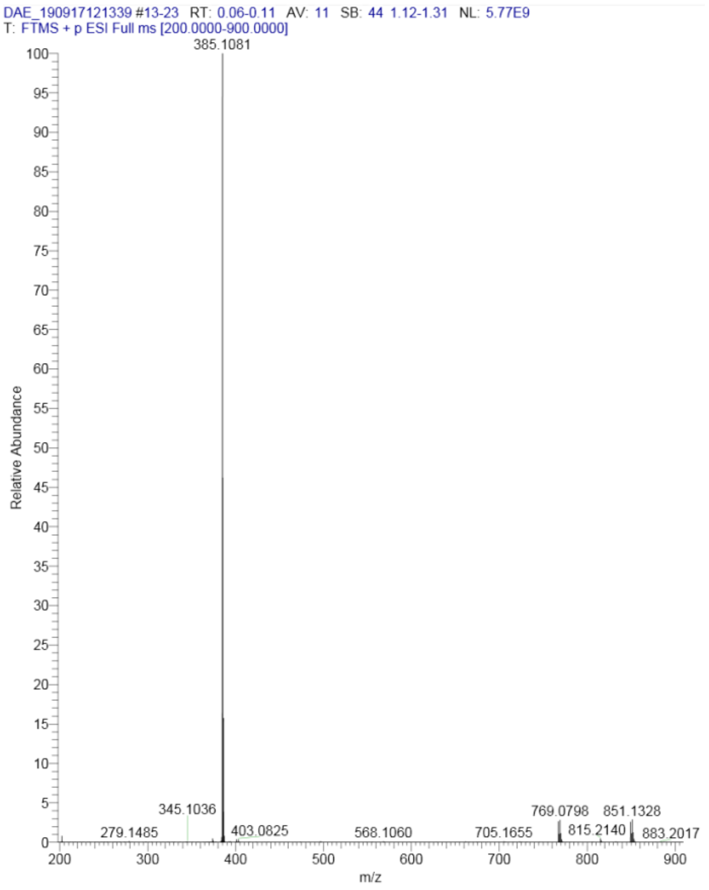

Supplementary Fig. 21 HRMS spectrum of compound OF-**1**.

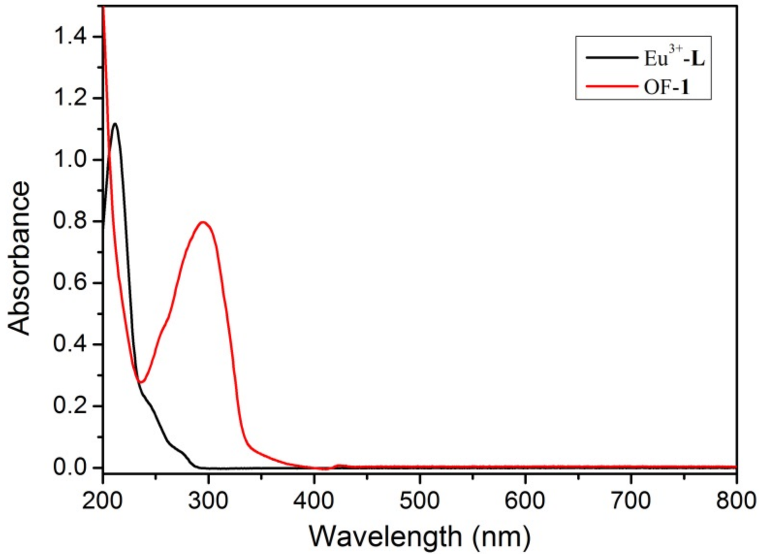

Supplementary Fig. 22 UV-Vis spectra of Eu^{3+}-**L** (black curve) and OF-**1** (red curve).

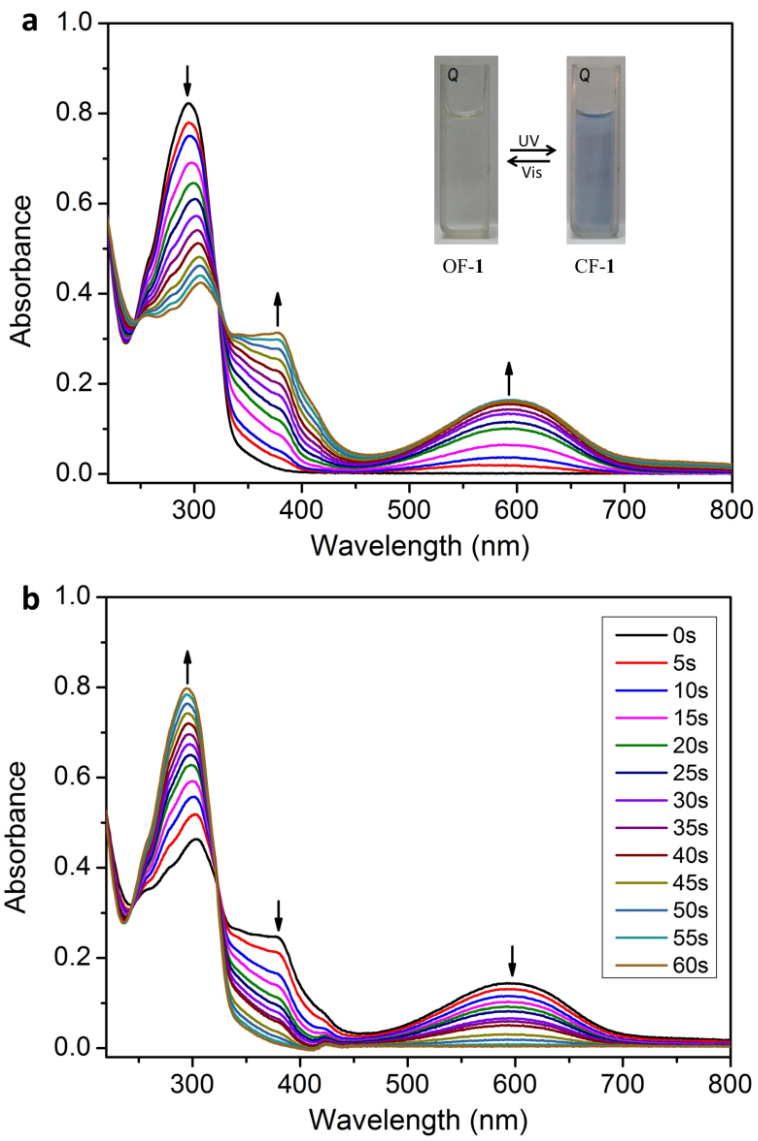

Supplementary Fig. 23 UV-Vis spectral changes. UV-Vis spectral changes of OF-**1** aqueous solution (2.1 × 10^{-5} M) upon the irradiation with (a) 300 nm UV light for up to 60s and (b) subsequent irradiation with visible light (>450 nm). Inset in (a): corresponding photographic images of **1** aqueous solution upon alternating UV and visible light irradiation.

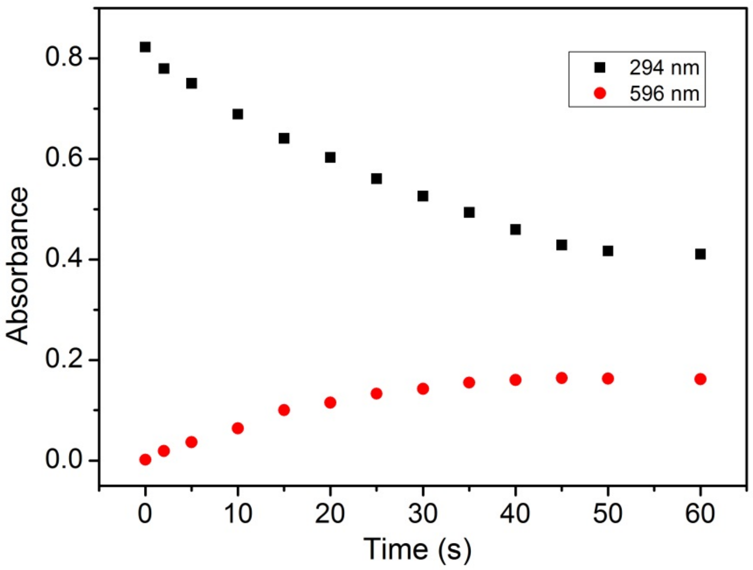

Supplementary Fig. 24 Absorbance changes of OF-**1** at 294 and 596 nm upon the irradiation with 300 nm UV light.

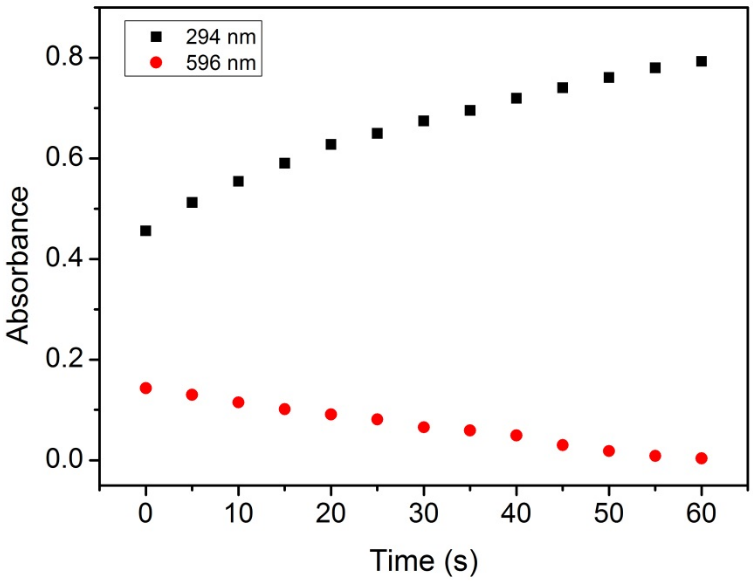

Supplementary Fig. 25 Absorbance changes of the resulted CF-**1** at 294 and 596 nm upon subsequent irradiation with >450 nm visible light.

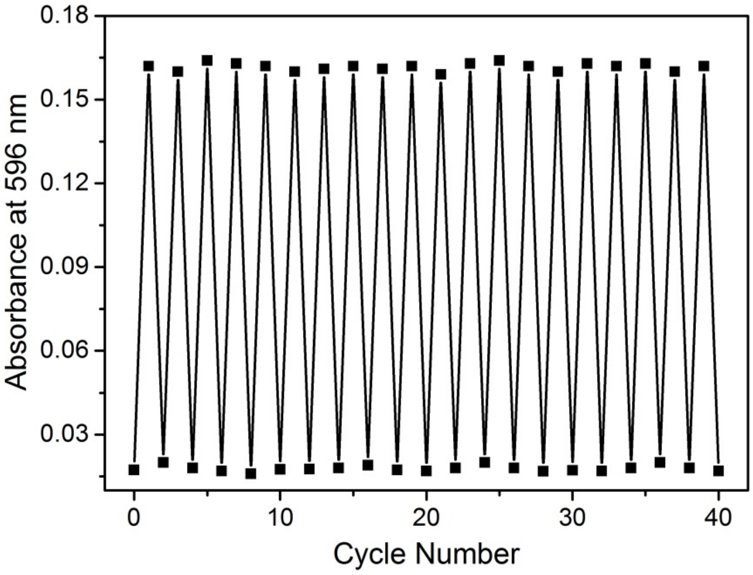

Supplementary Fig. 26 Absorbance intensity changes of compound **1** at 596 nm upon repeatedly alternating irradiation with UV and visible light.

Supplementary Note 1

As shown in Supplementary Fig. 23, the UV–Vis spectrum of OF-**1** showed a strong absorption at 294 nm ($\varepsilon = 3.9 \times 10^4$ cm^{-1} M^{-1}), and no absorption >400 nm was observed. Upon the irradiation with 300 nm UV light, the absorption band at 294 nm decreased gradually and new peaks appeared at 380 and 596 nm ($\varepsilon = 7.8 \times 10^3$ cm^{-1} M^{-1}) with an isosbestic point at 323 nm. These changes reached the photostationary state in 60 s (Supplementary Fig. 24), leading to an obvious color change of the solution from colorless to blue (inset of Supplementary Fig. 23a). A combination of these phenomena indicates the transition from OF-**1** to CF-**1**. Interestingly, a complete recovery to the original UV–Vis spectrum of OF-**1** was achieved upon subsequent irradiation of CF-**1** with visible light (λ > 450 nm) in 60 s, accompanied with the color change of blue solution back to colorless (Supplementary Figs. 23b and 25). Notably, this ring-open/ring-close photoisomerization cycle was repeatable for at least 20 times without apparent fatigue (Supplementary Fig. 26), suggesting the good reversibility of this process.

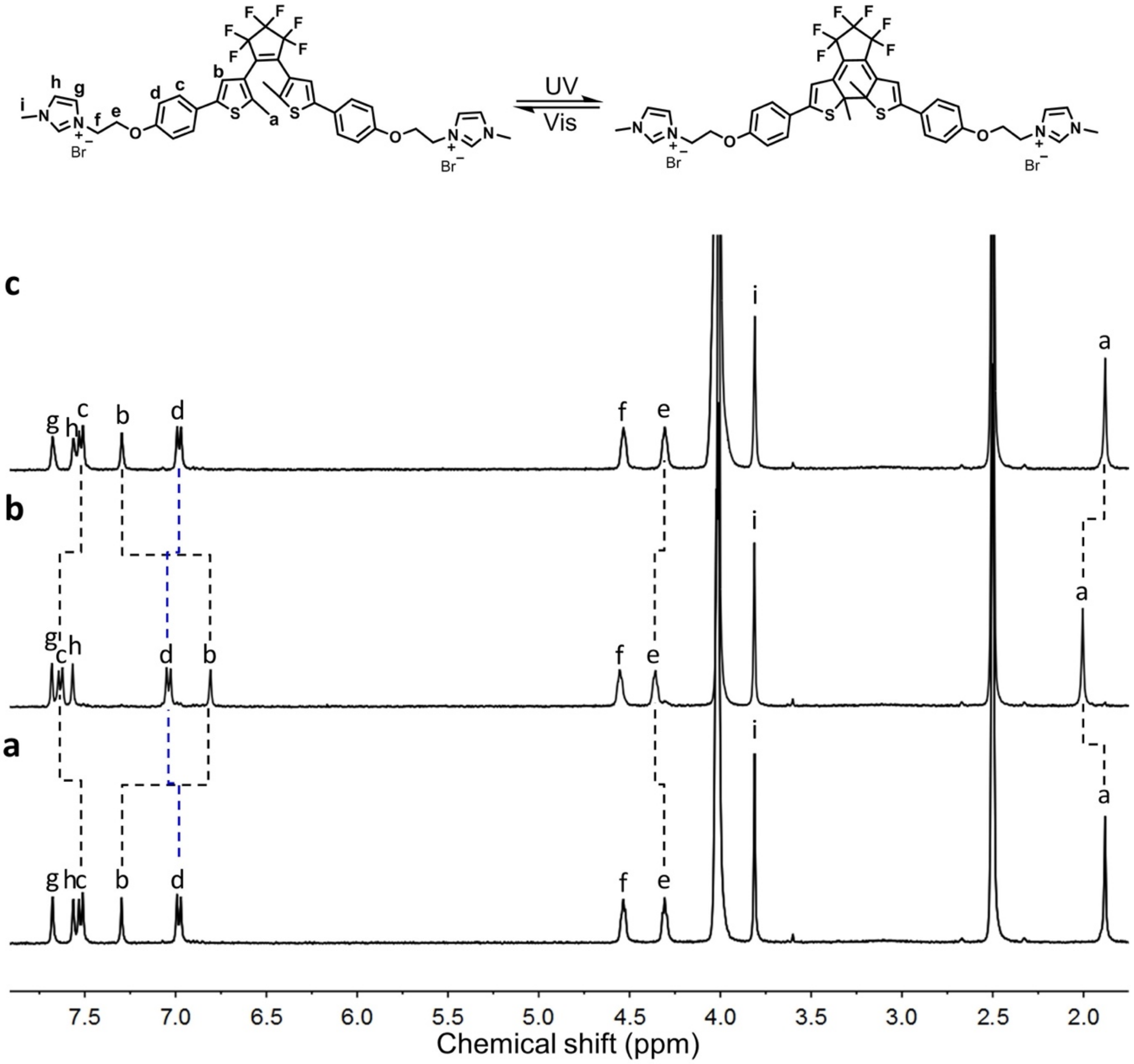

Supplementary Fig. 27 ^{1}H NMR spectral studies. Partial ^{1}H NMR spectra (DMSO:D_2O = 4:1, 400 MHz, 25 °C, $C_{\text{OF-}\mathbf{1}}$ = 2.1 × 10^{-4} M) of compound OF-**1** (a) before and (b) after irradiation with 300 nm UV light for 60 min, and (c) subsequent irradiation with visible light (>450 nm) for 60 min.

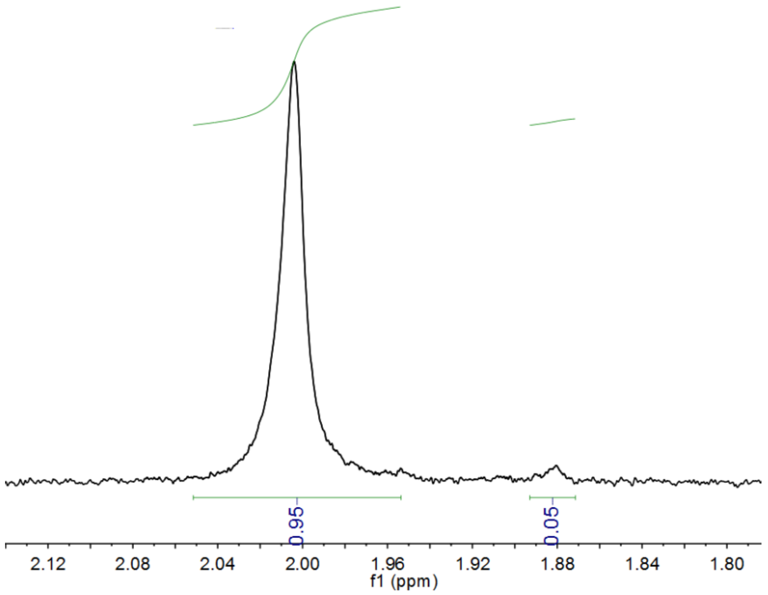

Supplementary Fig. 28 Amplified ^{1}H NMR spectrum (DMSO:D_2O = 4:1, 400 MHz, 25 °C) of OF-**1** after irradiation with 300 nm UV light for 60 min.

Supplementary Note 2

To further clarify the photoisomerization behavior of compound **1**, ^{1}H NMR spectra of OF-**1** in a mixed solvent were recorded (Supplementary Fig. 27). Upon 300 nm light irradiation, the methyl protons (H_a) showed a downfield shift from 1.88 ppm to 2.00 ppm, the thiophene protons (H_b) underwent a drastic upfield shift from 7.30 ppm to 6.81 ppm, and the aromatic protons (H_c and H_d) presented downfield shifts ($\Delta\delta$ = 0.11 ppm for H_c and 0.06 ppm for H_d). The molar ratio of CF-**1** : OF-**1** was determined to be 0.95:0.05 according to the integrating resonance of proton H_a (Supplementary Fig. 28), indicating nearly quantitative transition from OF-**1** to CF-**1** after 300 nm light irradiation. After subsequent irradiation with visible light, the CF-**1** isomer could be converted back to OF-**1**, as shown by the recovery to the original ^{1}H NMR spectrum (Supplementary Fig. 27c).

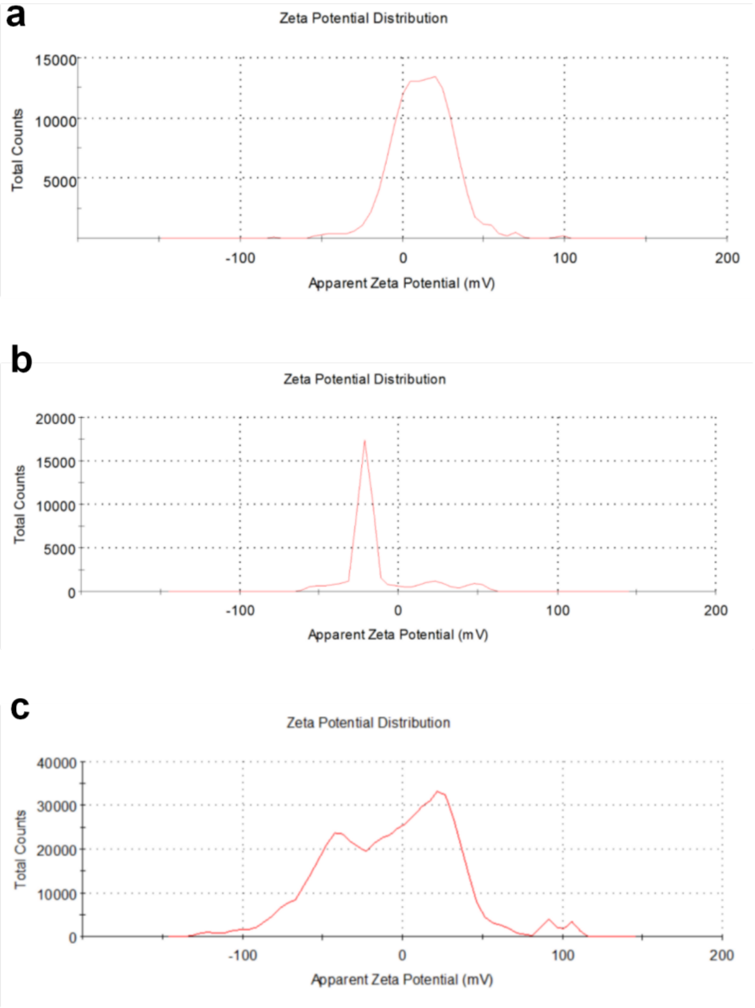

Supplementary Fig. 29 Zeta potential studies. Zeta potential of (a) OF-**1** (ζ = 20.15 mV), (b) Eu^{3+}-**L** (ζ = -19.53 mV), and (c) Eu^{3+}-**L**-OF-**1** (ζ = 1.54 mV).

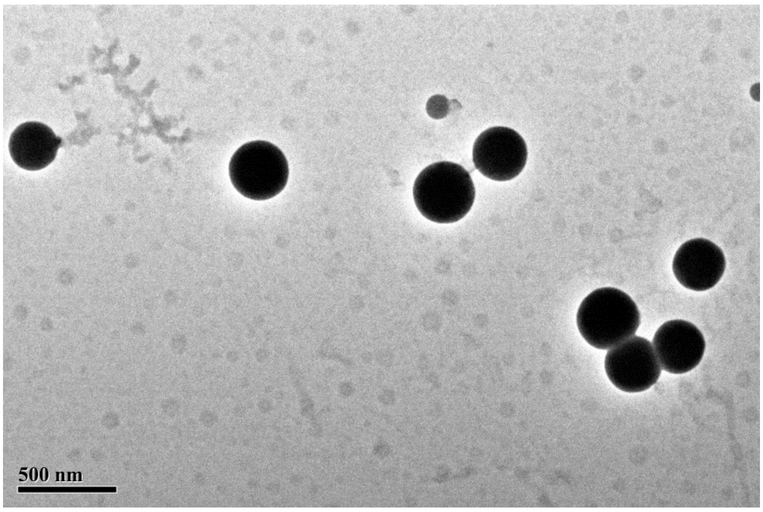

Supplementary Fig. 30 Transmission electron microscopy (TEM) image of Eu^{3+}-**L**-OF-**1**.

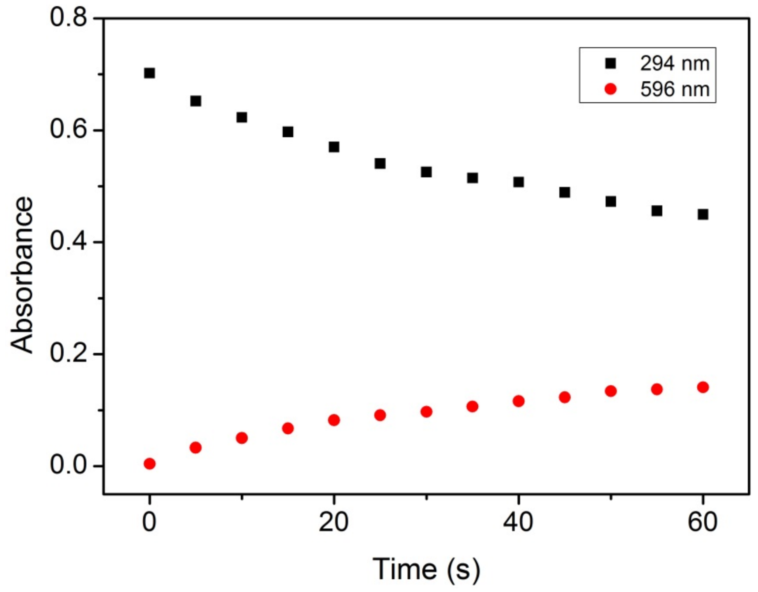

Supplementary Fig. 31 Absorbance changes of Eu^{3+}-**L**-OF-**1** at 294 and 596 nm upon irradiation with 300 nm UV light.

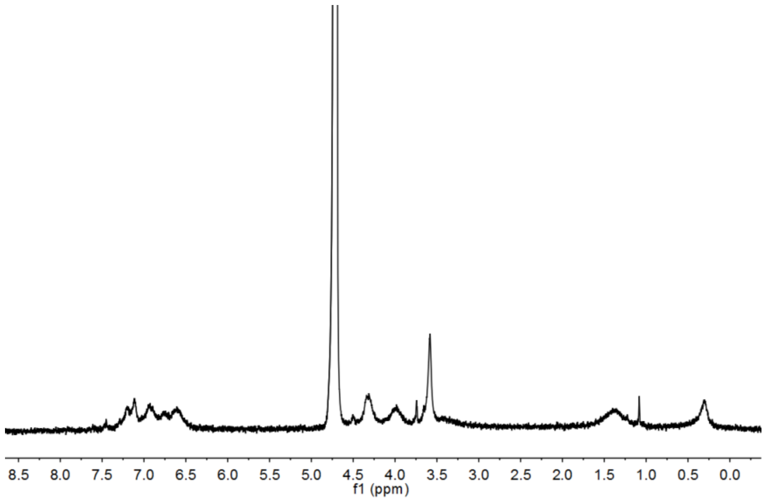

Supplementary Fig. 32 ^{1}H NMR spectrum of compound OF-**1** (D_2O, 400 MHz, 25 °C).

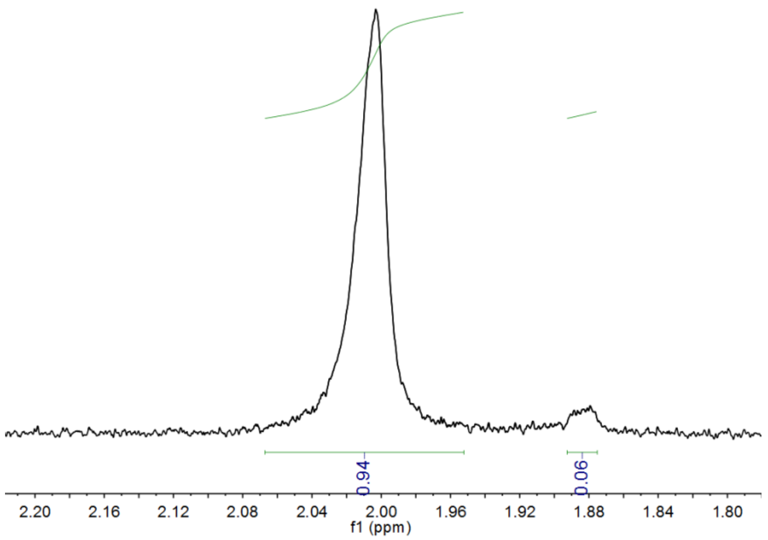

Supplementary Fig. 33 Amplified ^{1}H NMR spectrum of Eu^{3+}-**L**-CF-**1** (DMSO:D_2O = 4:1, 400 MHz, 25 °C).

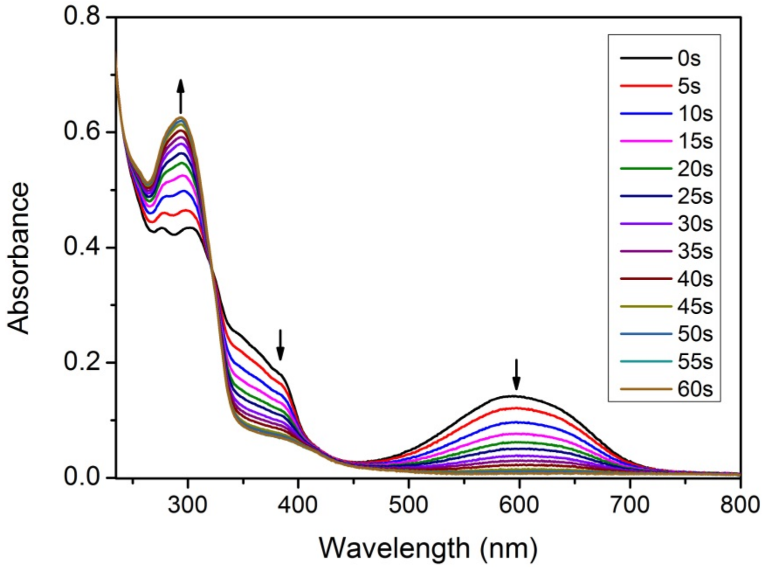

Supplementary Fig. 34 UV-Vis spectral changes of Eu^{3+}-**L**-CF-**1** aqueous solution ([Eu^{3+}] = 1.4×10^{-5} M, [**L**] = [CF-**1**] = 2.1×10^{-5} M) upon irradiation with visible light (>450 nm).

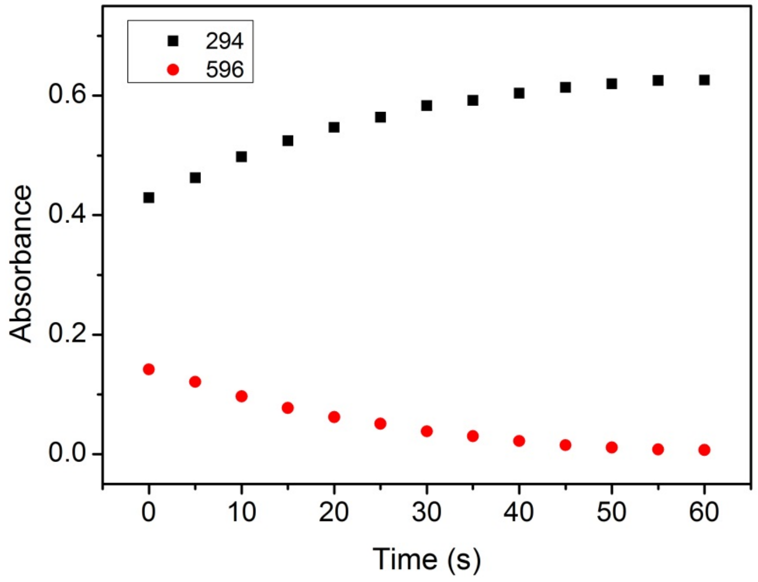

Supplementary Fig. 35 Absorbance changes of Eu^{3+}-**L**-CF-**1** at 294 and 596 nm upon irradiation with >450 nm visible light.

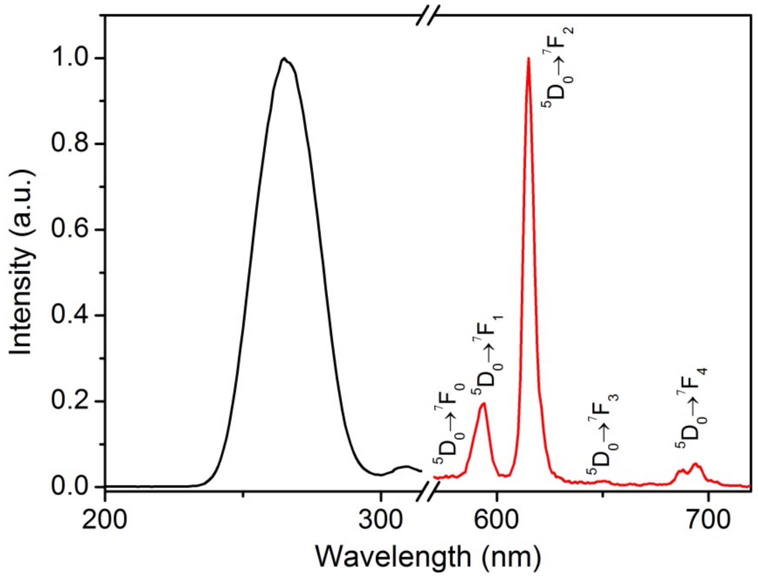

Supplementary Fig. 36 Normalized excitation (black, $\lambda_{em} = 615$ nm) and emission (red, $\lambda_{ex} = 265$ nm) spectra of Eu^{3+}-**L**-OF-**1**.

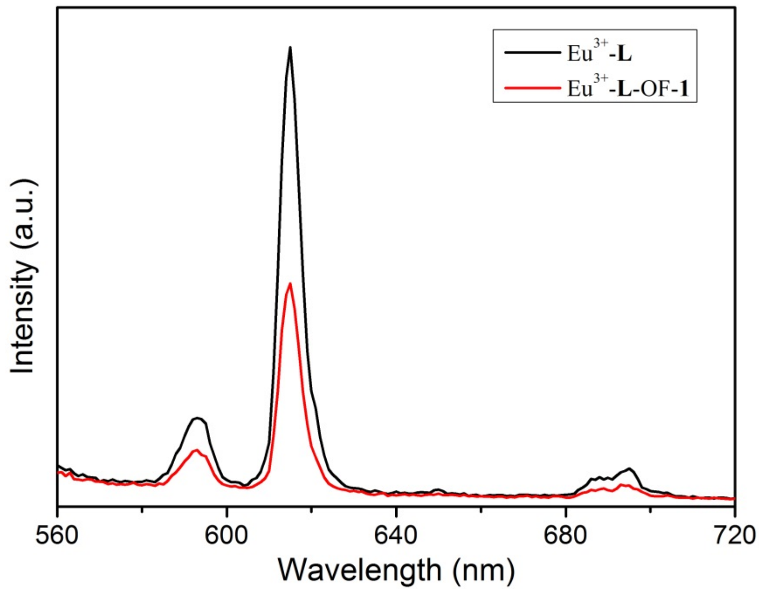

Supplementary Fig. 37 Luminescence emission of Eu^{3+}-**L** (black curve) and Eu^{3+}-**L**-OF-**1** (black curve) at the same concentration (λ_{ex} = 265 nm).

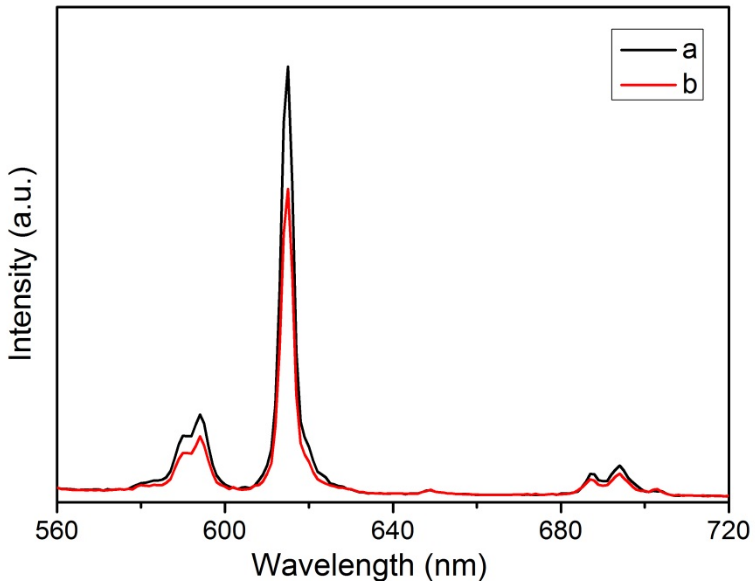

Supplementary Fig. 38 Luminescence emission studies. Luminescence emission intensity change of $Eu(DPA)_3$/compound **2** (1:1.5) mixture in EtOH (a) before and (b) after the irradiation with 300 nm UV light for 60s.

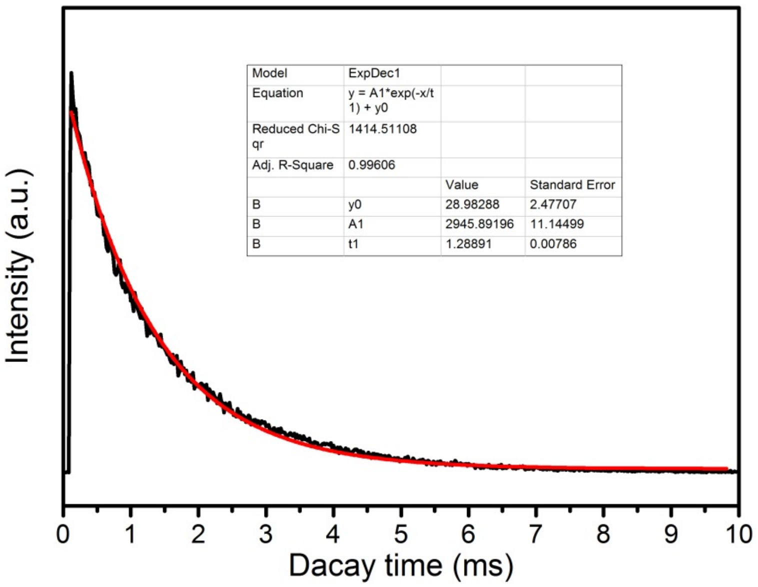

Supplementary Fig. 39 Luminescence decay curve of Eu^{3+}-**L**-OF-**1** (excited at 265 nm and monitored at 615 nm).

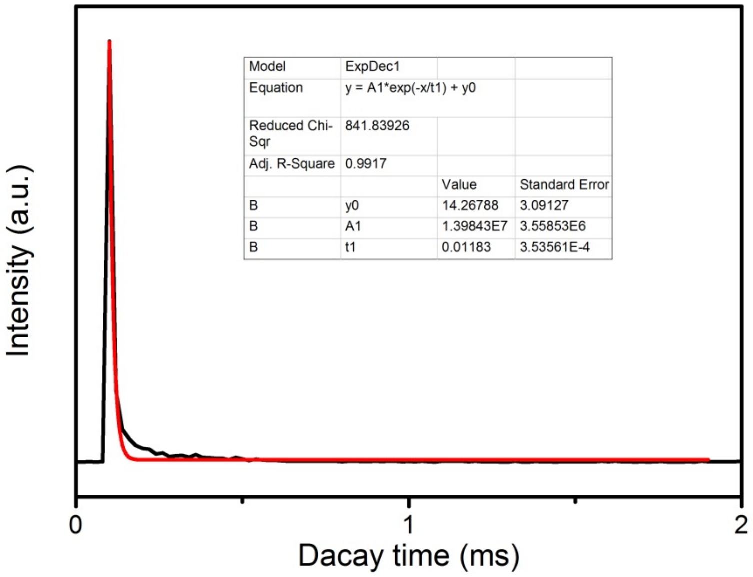

Supplementary Fig. 40 Luminescence decay curve of Eu^{3+}-**L**-CF-**1** (excited at 265 nm and monitored at 615 nm).

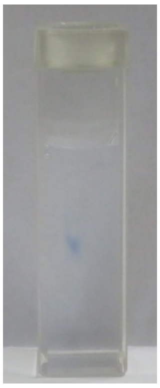

Supplementary Fig. 41 Photograph of Eu^{3+}-**L**-CF-**1** aqueous solution that was firstly irradiated with visible light (>450 nm) for 15 s, and then measured by luminescence analyzer (λ_{ex} = 265 nm).

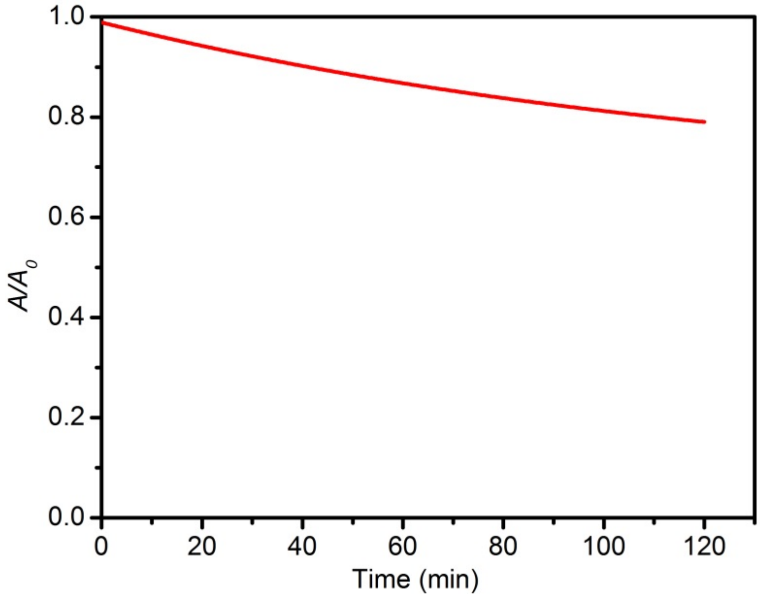

Supplementary Fig. 42 Absorption decay curve at 596 nm for Eu^{3+}-**L**-CF-**1** in aqueous solution at 25 °C.

Supplementary Table 1 First-order rate constants of Eu^{3+}-**L**-CF-**1** at different temperatures.

T/K	k/min^{-1}	$t_{1/2}$/min
293.15	0.001690	410.1
298.15	0.001840	376.7
303.15	0.002000	346.6
313.15	0.002380	291.2

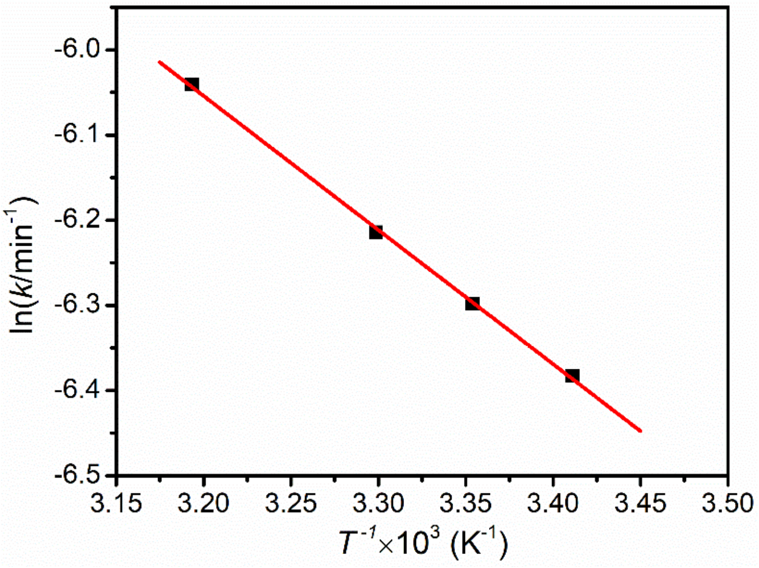

Supplementary Fig. 43 Temperature (*T*) dependence of the reaction rate constant (*k*).

Supplementary Note 3

The absorption decay of Eu^{3+}-**L**-CF-**1** at 596 nm obeys the first-order kinetics.[3-6] The kinetic equation is expressed by Lambert-Beer law:

$$\ln\frac{A}{A_0} = -kt \qquad (1)$$

where k is reaction rate constant, t is reaction time, and A_0 and A are absorbance of Eu^{3+}-**L**-CF-**1** at initial state (t = 0) and at random reaction time t, respectively. As shown in Supplementary Fig. 42, the absorption change (A/A_0) of Eu^{3+}-**L**-CF-**1** at 596 nm was recorded as a function of time (t) at 25 °C. The k and half-life ($t_{1/2}$) of Eu^{3+}-**L**-CF-**1** at 25 °C are 0.001840 min^{-1} and 376.7 min, respectively.

In addition, the relationship of k and T can be estimated by using Arrhenius equation:

$$\ln k = -\frac{E_a}{\mathrm{R}T} + \ln A \qquad (2)$$

where E_a is activation energy, R is gas constant, T is temperature, and A is frequency factor. Supplementary Fig. 43 shows the temperature dependence of k. E_a and A can be calculated by the slope and intercept of the linear plot. The E_a value of Eu^{3+}-**L**-CF-**1** was calculated as 13 kJ mol^{-1}.

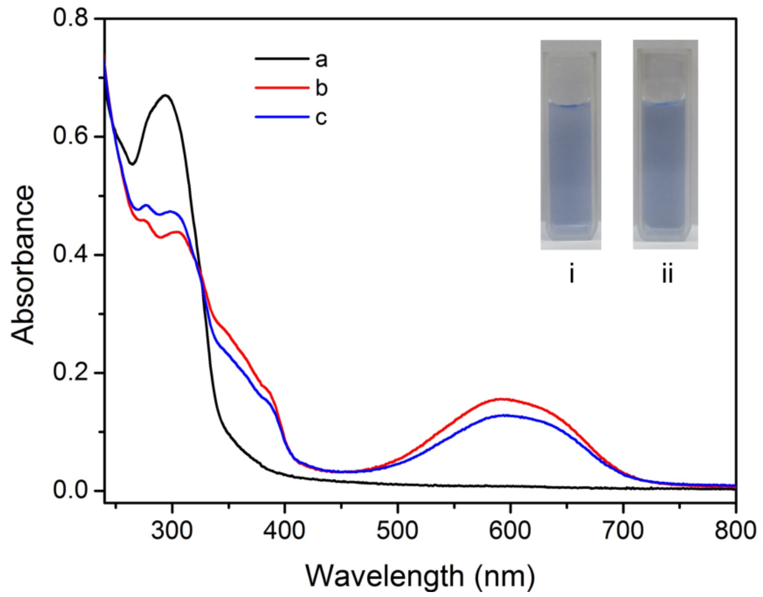

Supplementary Fig. 44 UV-Vis spectral studies. UV-Vis spectral changes of Eu^{3+}-**L**-OF-**1** (a) before and (b) after irradiation with 300 nm UV light for 60 s, and (c) then placing it under sunlight for 90 min. Inset shows the corresponding photographic images of Eu^{3+}-**L**-CF-**1** (i) before and (ii) after exposure to sunlight for 90 min.

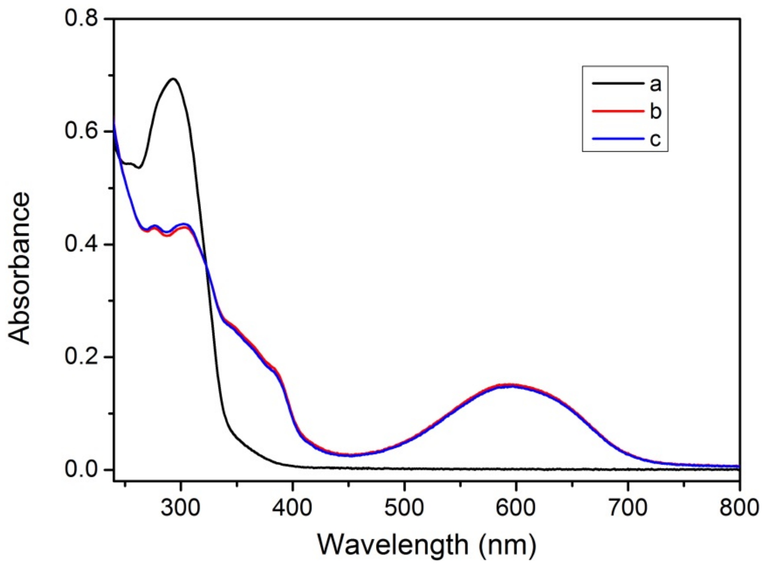

Supplementary Fig. 45 UV-Vis spectral studies. UV-Vis spectral changes of Eu^{3+}-**L**-OF-**1** (a) before and (b) after irradiation with 300 nm UV light for 60 s, and (c) then heated to 60 °C in the dark for 1 h.

Supplementary References

1. Li, B., Ding, Z.-J., Li, Z. & Li, H. Simultaneous enhancement of mechanical strength and luminescence performance in double-network supramolecular hydrogels. *J. Mater. Chem. C* **6**, 6869-6874 (2018).

2. Yin, X.-H., & Tan, M.-Y. Synthesis of novel multifunctional pyridine-2,6-dicarboxylic acid derivatives. *Synth. Commun*. **33**, 1113-1119 (2003).

3. Kitagawa, D., Nakahama, T., Nakai, Y. & Kobatake, S. 1,2-Diarylbenzene as fast T-type photochromic switch. *J. Mater. Chem. C* **7**, 2865-2870 (2019).

4. Nakahama, T., Kitagawa, D. & Kobatake, S. Tuning of optical properties and thermal cycloreversion reactivity of photochromic diarylbenzene by introducing electron-donating substituents. *J. Phys. Chem. C* **123**, 31212-31218 (2019).

5. Uchida, K. *et al.* Thermally reversible photochromic systems. Photochromism of a dipyrrolylperfluorocyclopentene. *Chem. Lett.* **8**, 835-836 (1999).

6. Irie, M., Lifka, T., Kobatake S. & Kato, N. Photochromism of 1,2-bis(2-methyl-5-phenyl-3-thienyl)perfluorocyclopentene in a single-crystalline phase. *J. Am. Chem. Soc.* **122**, 4871-4876 (2000).
