# Supplementary material for: Photoresponsive supramolecular coordination polyelectrolyte as smart anticounterfeiting inks
Source: Nat Commun. 2021 Mar 1;12:1363. doi: 10.1038/s41467-021-21677-4 (PMC7921134; doi:10.1038/s41467-021-21677-4)
Supplement: Supplementary file 3 — Description of Additional Supplementary Files [file 41467_2021_21677_MOESM3_ESM.pdf]

Description of Additional Supplementary Files

Title: Supplementary Movie 1

Description: This movie shows the scanning of the QR code by a smart phone under daylight. The QR code (5 × 5 cm) was prepared by printing Eu^{3+}-**L**-OF-**1** aqueous solution on a commercial blue PET film. The emitting color was invisible under daylight. As a consequence, the code was not recognized by the application software.

Title: Supplementary Movie 2

Description: This movie shows the scanning of the QR code by a smart phone under UV lamp. The QR code was exposed under a commercial UV lamp (254 nm) and scanned by smartphone. The QR code became visible and was easily recognized by the software, allowing read-out of the encoded information.

Title: Supplementary Movie 3

Description: This movie shows the scanning of the QR code after irradiation with UV light (300 nm). The QR code was irradiated with a 300W Xe lamp under specific wavelength (300 nm) for 1 min, and then scanned by the smartphone under daylight. The array was still invisible on blue PET film.

Title: Supplementary Movie 4

Description: This movie shows the scanning of the QR code after irradiation with UV light (300 nm) under UV lamp. The QR code was irradiated with a 300W Xe lamp under specific wavelength (300 nm) for 1 min, and then exposed under commercial UV lamp and scanned by the smartphone. The luminescence was quenched, accompanied with the disappearance of the luminescent array. As a result, the coding information became invisible and cannot be read out.

Title: Supplementary Movie 5

Description: The QR code was irradiated with a 300W Xe lamp under specific wavelength (300 nm) for 1 min, placed under daylight for one month, and then scanned by the smartphone under daylight. Because the emitting color was invisible, the coding information was still unreadable.

Title: Supplementary Movie 6

Description: The QR code was irradiated with a 300W Xe lamp under specific wavelength (300 nm) for 1 min, and placed under daylight for one month. Then, the pattern was exposed under commercial 254 nm UV lamp and scanned by the smartphone. The coding information was still unreadable.

Title: Supplementary Movie 7

Description: This movie shows the scanning of the QR code after 20 consecutive cycles of alternating exposure to UV and visible light. The QR code was exposed under 300 nm UV light for 1 min, and then irradiated with visible light (>450 nm) for 2 min. Repeating this process for 20 times, and then scanning by the smartphone under daylight were conducted. The information was unreadable because the emitting color was invisible under daylight.

Title: Supplementary Movie 8

Description: This movie shows the scanning of the QR code after 20 consecutive cycles of alternating exposure to UV and visible light. The QR code was exposed under 300 nm UV light for 1 min, and then irradiated with visible light (>450 nm) for 2 min. Repeating this process for 20 times, and then scanning by the smartphone under commercial UV lamp were conducted. The information can be read out repeatedly without any hysteresis.
